# Spatial analyses revealed CXCL5 and SLC6A14 as the markers of microvascular invasion in intrahepatic cholangiocarcinoma

**DOI:** 10.1097/HC9.0000000000000597

**Published:** 2024-12-11

**Authors:** Guangyu Fan, Liyuan Dai, Tongji Xie, Lin Li, Le Tang, Xiaohong Han, Yuankai Shi

**Affiliations:** 1 Department of Medical Oncology, National Cancer Center/National Clinical Research Center for Cancer/Cancer Hospital, Chinese Academy of Medical Sciences & Peking Union Medical College, Beijing Key Laboratory of Clinical Study on Anticancer Molecular Targeted Drugs, Chaoyang District, Beijing, China; 2 Department of Clinical Laboratory, National Cancer Center/National Clinical Research Center for Cancer/Cancer Hospital, Chinese Academy of Medical Sciences & Peking Union Medical College, Beijing Key Laboratory of Clinical Study on Anticancer Molecular Targeted Drugs, Chaoyang District, Beijing, China; 3 Department of Pathology, National Cancer Center/National Clinical Research Center for Cancer/Cancer Hospital, Chinese Academy of Medical Sciences & Peking Union Medical College, Beijing, China; 4 Department of Clinical Pharmacology Research Center, Peking Union Medical College Hospital, State Key Laboratory of Complex Severe and Rare Diseases, NMPA Key Laboratory for Clinical Research and Evaluation of Drug, Beijing Key Laboratory of Clinical PK & PD Investigation for Innovative Drugs, Chinese Academy of Medical Sciences & Peking Union Medical College, Dongcheng District, Beijing, China

**Keywords:** immunosuppression, intrahepatic cholangiocarcinoma, microvascular invasion, spatial transcriptomics

## Abstract

**Background:**

Microvascular invasion (MVI) is a critical prognostic factor in intrahepatic cholangiocarcinoma (ICC), strongly associated with postoperative recurrence. However, the phenotypic features and spatial organization of MVI remain inadequately understood.

**Methods:**

We performed a spatial transcriptomic analysis on 29,632 spots from six ICC samples, manually delineating MVI clusters using the cloupe software. Key biomarkers were identified and validated in an independent cohort of 135 ICC patients. Functional and survival analyses were conducted to assess clinical relevance, and cell-cell communication pathways were investigated.

**Results:**

MVI regions exhibited heightened proliferation, angiogenesis, and epithelial-mesenchymal transition, driven by increased expression of transcription factors SOX10, ZEB1, and SNAI2. CXCL5 and SLC6A14 were identified as potential MVI biomarkers and showed high expression in tumor-invasive areas. Serum CXCL5 demonstrated strong predictive power for vascular invasion (AUC = 0.92) and intrahepatic metastasis (AUC = 0.96). High expression of both CXCL5 and SLC6A14 was associated with the worst survival outcomes. MVI regions were enriched with immunosuppressive MRC1+ macrophages and exhibited elevated immune checkpoint expression, including HAVCR2 and TIGHT, indicative of immune resistance. Cell-cell communication analysis revealed CXCL5-CXCR2 and LGALS9-HAVCR2 as key ligand-receptor pairs contributing to the immunosuppressive microenvironment.

**Conclusions:**

This study identifies CXCL5 and SLC6A14 as key biomarkers of MVI, highlighting their roles in tumor proliferation, immune resistance, and poor clinical outcomes. These findings provide valuable insights into the spatial organization of MVI and its contribution to ICC progression, offering potential therapeutic targets.

## INTRODUCTION

Intrahepatic cholangiocarcinoma (ICC), the second most prevalent primary hepatic cancer, is associated with a disappointing 5-year survival rate of <20%.^1^,^2^,^3^ Moreover, despite the frustratingly limited treatment options, the global prevalence of ICC is expected to increase by up to 10-fold over the next 2–3 decades.^4^ Alarmingly, postoperative recurrence is common among patients, with a 5-year recurrence incidence reaching as high as 50% after resection.^5^ While surgical procedures can effectively manage early-stage ICC, there is an urgent need to elucidate the underlying processes driving recurrence, identify high-risk individuals, and implement interventions to mitigate the associated morbidity and mortality.

Microvascular invasion (MVI) is the infiltration of cancer cells into small blood vessels that are lined with endothelial cells. This invasion can only be detected through the use of a microscope. MVI is closely linked to the aggressive behavior of tumors and the occurrence of distant metastasis. In addition, it is a crucial predictive factor for both mortality and recurrence of ICC, as supported by studies. Research has demonstrated that the rate of recurrence in patients with MVI is twice as high as in those without MVI.^6^ Furthermore, there is a suggestion that MVI plays a significant role in the pathological progression of intrahepatic metastases in ICC.^7^ Nevertheless, evaluating MVI requires thoroughly examining the complete surgical specimen under a microscope, which makes it less feasible as a predictive tool in clinical decision-making, despite its clear clinical importance. Therefore, it is evident that there is a significant requirement for the identification of biomarkers to detect MVI. Furthermore, understanding the specific molecular factors that cause MVI in ICC could aid in the discovery of new treatment targets and ultimately result in better patient outcomes.

Data obtained from conventional bulk-level transcriptomics are incapable of distinguishing MVI from other types of cancer cells. Single-cell RNA sequencing fails to consider spatial context, thereby hindering the investigation of connections between the local environment and specific cell-cell interactions. Recent advancements in spatial transcriptomics technology have introduced powerful tools for profiling the precise spatial distribution of genes and understanding how the intrinsic characteristics of tumors interact with other crucial cell types within the context of tumor growth and therapeutic response.^8^ Spatial transcriptomics facilitates the analysis of the molecular and cellular composition of MVI, as well as its interactions with neighboring components of the tumor microenvironment (TME) by the preservation of tissue architecture.^9^

In this study, we used spatial transcriptomics technology to characterize the phenotype of MVI and its surrounding TME in ICC. We identified CXCL5 and SLC6A14 as potential indicators for MVI. Furthermore, in a substantial in-house cohort comprising 135 patients, we validated CXCL5 as a serum marker for MVI. Notably, our research revealed that MVI and its precursor cells have the capacity to recruit MRC1+ tumor-associated macrophages (TAMs) while excluding CD8 T cells. In addition, we observed elevated expression of numerous immune checkpoints, including HAVCR2 and TIGHT, around MVI, suggesting an immune-resistant microenvironment. In summary, our findings not only elucidate the roles of CXCL5 and SLC6A14 as indicators of MVI but also provide insights into the unique spatial architectures of MVI that facilitate the development of immunological resistance.

## METHODS

### Patient samples

Formalin-fixed paraffin-embedded ICC samples were collected from 70 patients who were under treatment at the Cancer Hospital, Chinese Academy of Medical Science in Beijing, China. This was done in accordance with the ethical procedures established by the institution, and patients gave their informed consent before the samples were collected. The protocol was allowed to proceed after being approved by the Ethics Committee of the Institute Curie (no. 23/262-4004). Pathologists are responsible for determining the stage of each sample according to the American Joint Committee on Cancer.

### Data and materials

Data from single cells were acquired from the Gene Expression Omnibus (GEO) database with the accession numbers GSE125449, GSE138709, GSE151530, and GSE181878.^10^,^11^,^12^,^13^ The clinical data and information were obtained from the original studies. This study analyzed 21,158 cells from 35 ICC tumor samples based on these data sets. The bulk transcriptomics data and clinical characteristics of the FU-iCCA cohort were collected from a published study with 252 patients with ICC.^14^

### Clustering analysis of spatial transcriptomics

The spatial transcriptomics data were subjected to clustering analysis using Seurat, with parameters manually curated to ensure an optimal classification of cell types based on empirical information. Spots with low quality, defined as having fewer than 500 identified genes or more than 6000 genes, and those with over 20% mitochondrial counts, were excluded during the data preparation stage. The SCT normalization algorithm from Seurat was used to normalize gene expression. Principal component analysis–based dimension reduction was conducted for downstream grouping and visualization. Twenty main components were collected for Louvain clustering to characterize cell types, with a resolution of 0.8.

### Differential expression analysis and gene set enrichment analysis

Differentially expressed gene (DEG) analysis was conducted in each cluster using the FindAllMarkers function from the Seurat package, with min.pct set to 0.1 and logfc.threshold set to 0.25. Pathway analysis was performed using the R package fgsea to clarify the biological functions of the detected DEGs in each cluster. The investigation involved assessing the enrichment of gene sets, including cancer hallmark, Biological Process Gene Ontology, and Kyoto Encyclopedia of Genes and Genomes.

### Dimension reduction and clustering analysis for single-cell data

The top 2000 most variable genes were identified using the FindVariableFeatures function and then used for principal component analysis within the Seurat package. We used the Harmony algorithm from the Harmony R package to address batch effects in the data sets before doing the clustering analysis. Cell subtypes were distinguished using the FindNeighbors and FindCluster algorithms. Cells were labeled with specific markers: epithelial cells (EPCAM, KRT8, and KRT19), fibroblasts (COL1A1, COL1A2, and DCN), endothelial cells (PLVAP, VWF, and PECAM1), T cells (CD3D, CD3E, and TRAC), B cells (MS4A1, CD79A, and CD79B), and myeloid cells (CD14, CD163, and CD68).

### Transcription factor analysis

The activity of transcription factors (TFs) was determined by using the Dorothea database, which includes documented TF-target interactions. We used the “dorothea regulon human” wrapper function from the DoRothEA package to build TF regulons, selecting high-confidence TFs categorized as levels “A,” “B,” and “C.” We used the VIPER technique in conjunction with the DoRothEA package by employing the run_viper function to calculate TF activity based on the Dorothea regulons.

### Survival analysis

For survival analyses, patient tissue samples were categorized into high and low groups. This classification was performed using the surv_cutpoint function in the R survminer package. Kaplan-Meier survival curves were generated to compare the different groups and evaluate the impact of specific genes on patient prognosis by assessing the proportions of patients surviving for a specified period. The log-rank test was employed to determine the statistical significance of the observed differences.

### CARD

The CARD algorithm from the R package CARD was employed to predict the cell composition of 6 primary cell types (epithelial cells, myeloid cells, fibroblasts, endothelial cells, T cells, and B cells).^15^ This algorithm integrates cell-type–specific expression information from scRNA-seq with correlations in cell-type composition across tissue locations. The initial step involved preparing the input data, including scRNA-seq data for the 6 main cell types and spatial transcriptomics data. This was accomplished using the createCARDObject function. Subsequently, the CARD_deconvolution function was applied to predict the cell composition of each spot in the spatial transcriptomics data. Finally, the CARD.visualize.pie function was employed to visualize the cell proportion of the 6 main cell types in each spot.

### Cell-cell communication analysis

CellChat was employed to analyze communication relationships and identify signaling molecules. CellChatDB.human was used to examine the primary signaling inputs and outputs across all cell clusters.

### Statistical analysis

The Mann-Whitney *U* test was conducted to assess the disparities between the 2 groups. The Spearman correlation test was used to evaluate the relationships between 2 variables. A 2-tailed *p* value of 0.05 was considered statistically significant. R version 4.1.0 was used for all data processing, statistical analysis, and graphing tasks.

## RESULTS

### The phenotype of MVI

**FIGURE 1:**
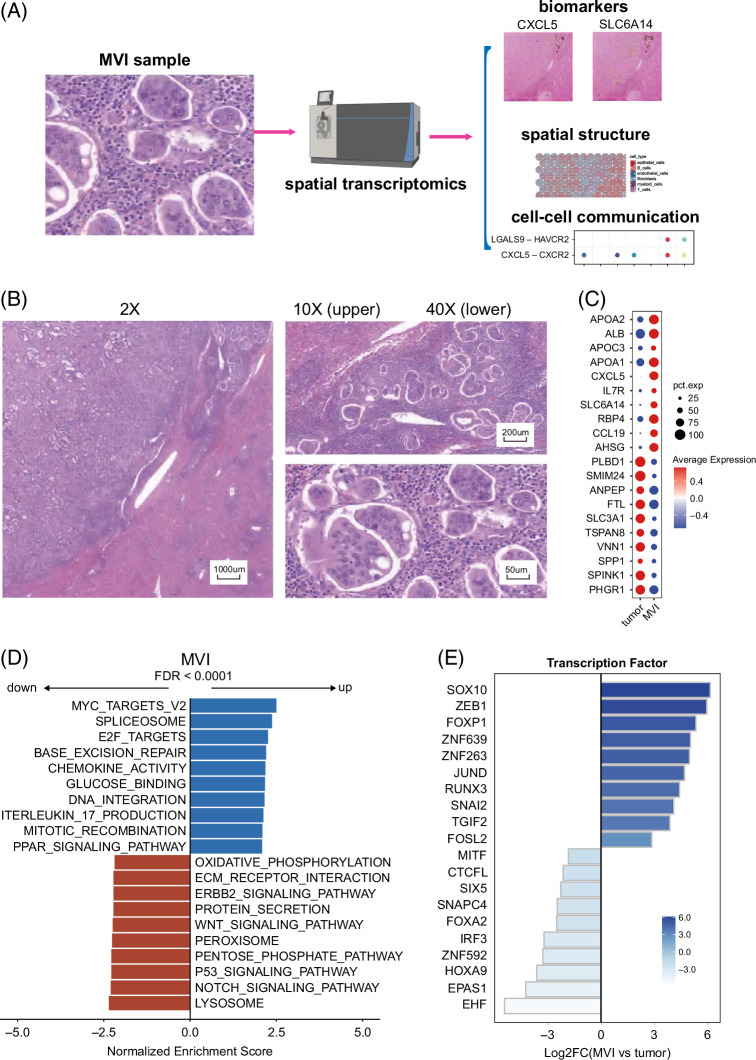
The phenotype of MVI in spatial transcriptomics data. (A) The methodology employed in this study. (B) The ×2, ×10, and ×40 HE images of the MVI, revealing multiple clusters of MVI in close proximity to the tumor boundary. (C) The top 10 upregulated and downregulated DEGs of MVI. (D) Bar chart displaying the 10 upregulated and 10 downregulated pathways in MVI. (E) Expression patterns of the 20 most varied TFs in MVI. Abbreviations: DEG, differentially expressed gene; HE, hematoxylin and eosin; MVI, microvascular invasion; TF, transcription factor.

The methodology employed in this study is depicted in Figure 1A. MVI stands as a potent, validated, and independent predictor of early recurrence and diminished survival time following surgical treatment for ICC.^16^ In this investigation, we obtained 6 formalin-fixed paraffin-embedded samples from patients with ICC, with one of these samples exhibiting the MVI. Through this specimen, we conducted a comprehensive exploration of potential biomarkers and the TME characteristics associated with MVI.

Initially, within the hematoxylin and eosin image of the MVI sample, we identified multiple clusters of MVI in close proximity to the ICC tumor boundary, accompanied by notable recruitment of a substantial number of immune cells surrounding the MVI (Figure 1B). Within each MVI cluster, a conglomeration of tumor cells was observed to accumulate within the microvascular lumina. Leveraging the advantages of spatial transcriptomics, which seamlessly integrates RNA expression data with spatial information, we accurately located these MVI clusters and acquired their gene expression profiles.

To identify potential MVI markers, we initially manually delineated the MVI clusters and tumor regions using the 10X-developed cloupe software. By comparing the gene expression profiles of MVI clusters and other tumor regions, we compiled a list of DEGs (Figure 1C, Supplemental Table S1, http://links.lww.com/HC9/B158). Among these DEGs, SLC6A14, a transporter responsible for mediating the influx of glutamine, serine, glycine, and methionine into cancer cells, displayed heightened expression in MVI.^17^ Notably, CXCL5 and CCL19 were 2 chemokines found to be highly expressed in MVI, with CXCL5 having previous associations with metastasis in colorectal cancer and breast cancer. In addition, APOA2, APOA1, APOC3, and RBP4, which encode lipid-related proteins, exhibited upregulation in MVI.^18^

Pathway analysis unveiled increased proliferation activities in MVI compared to tumor regions, characterized by upregulation of MYC, E2F, spliceosome, and mitotic recombination pathways (Figure 1D). Active chemokine activity and IL-17 production were observed in MVI, suggesting a potential immuno-modulatory role. In the meanwhile, there was suppressed involvement in the lysosome, NOTCH signaling, P53 signaling, and WNT signaling pathways in MVI.

Furthermore, we scrutinized the role of TFs in promoting the aggressive phenotype of MVI (Figure 1E). We used Dorothea to investigate potential discrepancies in regulon activity between MVI and other tumor areas. The expression patterns of the 20 TFs with the most diverse activity in cellular populations are depicted. Noteworthy findings included enhanced regulon activities of SOX10, which plays an essential role in tumor proliferation, migration, and apoptosis.^19^ In addition, ZEB1 and SNAI2, well-known epithelial-mesenchymal transition TFs, exhibited heightened regulon activities in MVI.^20^ FOXP1 also displayed increased activities and is associated with poor prognosis in diffuse large B-cell lymphoma and HCC.^21^,^22^

### SLC6A14 and CXCL5 were biomarkers of MVI

**FIGURE 2:**
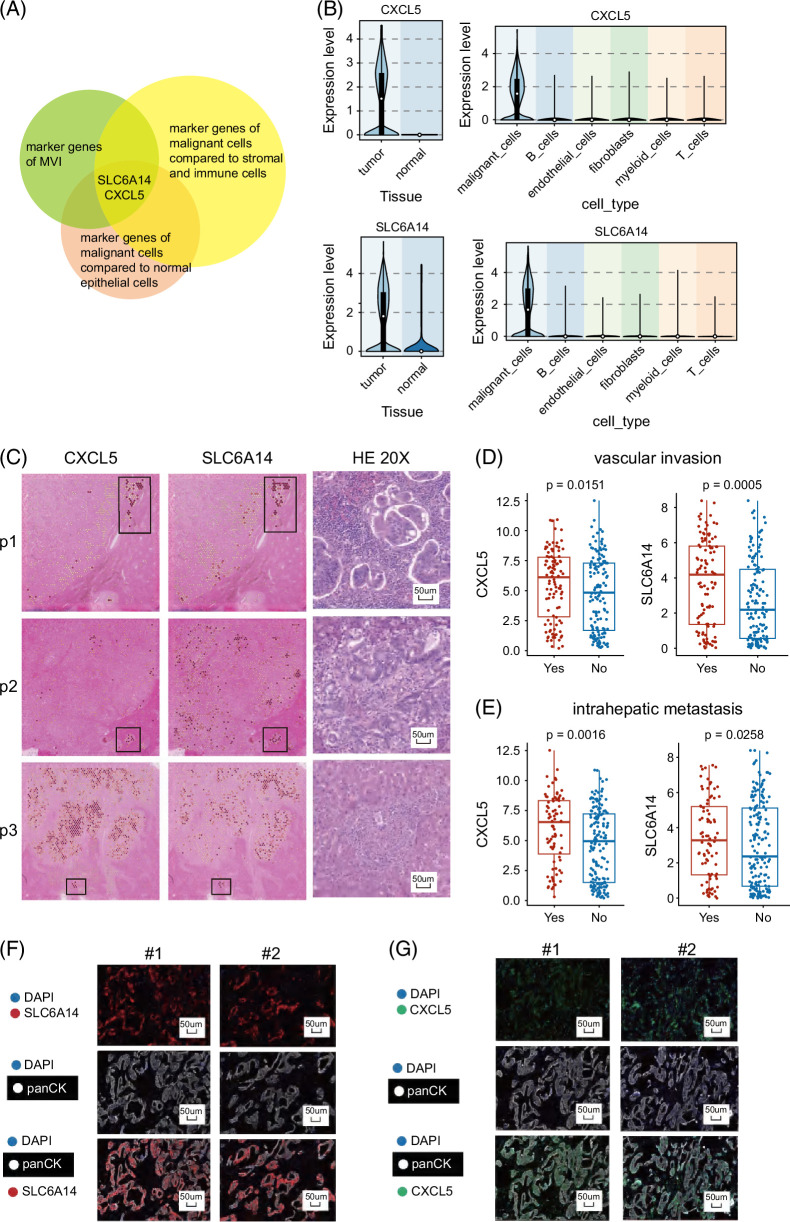
SLC6A14 and CXCL5 were biomarkers of MVI. (A) SLC6A14 and CXCL5 were identified as the potential MVI markers. (B) SLC6A14 and CXCL5 exhibited elevated expression in tumor cells compared to normal epithelial cells, immune cells, and stromal cells. (C) SLC6A14 and CXCL5 showed notable expression within MVI clusters (p1) and in tumor clusters invading normal regions (p2 and p3). (D) SLC6A14 and CXCL5 exhibited high expression in the vascular invasion group (n = 148) in a substantial cohort of patients with ICC (n = 255) with bulk transcriptomics data. (E) SLC6A14 and CXCL5 exhibited high expression in the intrahepatic metastasis group (n = 87) in a substantial cohort of patients with ICC (n = 255) with bulk transcriptomics data. (F) The multiplex immunofluorescence (multiplex immunofluorescence) performed in 20 patients demonstrated the main expression of SLC6A14 in tumor cells. (G) The multiplex immunofluorescence performed in 20 patients demonstrated the main expression of CXCL5 in tumor cells. Abbreviation: MVI, microvascular invasion.

To discover possible biomarkers for MVI, we began by obtaining DEGs between MVI and other tumor locations. We used the criteria of an average log2 fold change greater than 1 and a *p* value <0.05. Our hypothesis posited that the most suitable indicators for MVI should exhibit unique expression in tumor cells. Hence, we used the criterion of average log2 fold change >0.25 and *p* value <0.05 to identify marker genes that are exclusively expressed in tumor cells when compared to immune cells and normal epithelial cells. This analysis was performed using comprehensive single-cell data sets, including GSE125449, GSE138709, GSE151530, and GSE181878.^10^,^11^,^12^,^13^ Figure 2A illustrates the identification of 2 potential MVI biomarkers, SLC6A14 and CXCL5, through the intersection of these 3 gene lists. These genes exhibited elevated expression in tumor cells compared to immune cells and normal epithelial cells (Figure 2B).

We subsequently examined the spatial expression patterns of these genes (Figure 2C). In the MVI sample (p1), SLC6A14 and CXCL5 showed the highest expression within MVI clusters. In 2 samples (p2 and p3) featuring small tumor clusters invading normal regions, these genes also exhibited notable expression, indicating their invasive characteristics. Conversely, in the remaining 3 samples without metastasis tendencies, SLC6A14 and CXCL5 displayed low expression levels (Supplemental Figure S1, http://links.lww.com/HC9/B158). Furthermore, using a substantial cohort of patients with ICC with bulk transcriptomics data, of which 148 displayed vascular invasion out of 255 patients, we observed that both SLC6A14 and CXCL5 exhibited high expression in the vascular invasion group^14^ (Figure 2D). In addition, these 2 genes exhibited associations with intrahepatic metastasis (Figure 2E). To validate SLC6A14 and CXCL5 at the protein level, we performed multiplex immunofluorescence on 20 patients with ICC, using panCK to annotate tumor cells. SLC6A14 and CXCL5 demonstrated the co-location with panCK, indicating them as specific tumor markers (Figures 2F, G).

### MVI progenitors were identified based on similar copy number variation patterns

In tumors, genetic mutations accumulate over time, leading to the development of subpopulations of cells with distinct genetic profiles. The similarity of copy number variation (CNV) patterns could reflect the clonal evolution of tumor cells. The inferCNV analysis could specifically delineate the similarity of malignant cells based on their CNV patterns. To further trace the origin of MVI tumor cells, we conducted the inferCNV analysis on the MVI sample. This process involved 2 clustering phases.

**FIGURE 3:**
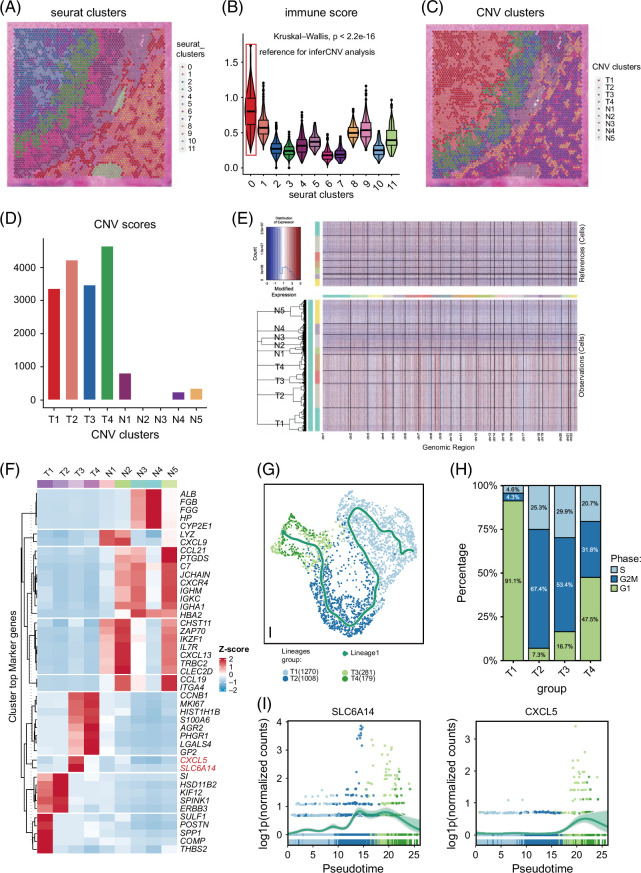
MVI progenitors were identified based on similar CNV patterns. (A) The identification of malignant cells in spatial transcriptomics data. (A) Clustering of 4647 spots into 12 distinct clusters. (B) Distribution of immune score in the 12 clusters. (C) Hierarchical clustering assigns all spots, except the reference cluster, into 8 clusters. (D) Bar charts showing the distribution of CNV score in all clusters. (E) The CNV patterns in the malignant clusters and the normal areas. (F) The expression profiles of the top 5 marker genes for each cluster. (G) Pseudotime ordering of all tumor cells organized these 4 tumor clusters into a single main branch. (H) The cell cycle phase of each tumor cluster, including phases G2M, G1, and S. (I) The transcriptional trajectories of SLC6A14 and CXCL5. Abbreviations: CNV, copy number variation; MVI, microvascular invasion.

The main goal of the first stage was to find reference cells for the inferCNV pipeline. Afterward, the inferCNV analysis used these reference cells to deduce the CNV patterns of malignant cells. Using the Seurat software, the 4647 points in this sample were divided into 11 separate clusters (Figure 3A). An “immune score” was calculated for each spot based on a set of genes relevant to the immune system. The genes included universal immunity markers (PTPRC), universal T-cell markers (CD2, CD3D, CD3E, and CD3G), B-cell markers (CD79A, MS4A1, and CD79B), and myeloid cell markers (CD68 and CD14). The “immune score” represents the mean value of immune infiltration within each spot. The reference cluster for the inferCNV study was chosen to be cluster 1 from the original clustering analysis since it had the greatest immunological score (Figure 3B).

The primary objective of the second clustering phase was to differentiate malignant cells from other cell types by analyzing CNV patterns. After performing hierarchical clustering using tree partitioning, all sites, except for the reference cluster, were classified into 8 groups based on their CNV patterns (Figure 3C). Clusters T1, T2, T3, and T4, which had high CNV values, were determined to be malignant clusters. In contrast, the other clusters had much lower CNV ratings (Figure 3D). Significant CNV patterns were seen in the malignant clusters when compared to the normal areas (Figure 3E). We corroborated these interpretations by consulting 2 pathologists who examined the hematoxylin and eosin histopathology material. According to the CNV analysis, clusters T1, T2, T3, and T4 represented tumor areas that were spread out. The remaining clusters were mainly composed of normal hepatocytes, stromal cells, and immune cells. The T1, T2, T3, and T4 subclusters were divided based on their distinctive CNV patterns using the inferCNV analysis. In the MVI-positive sample, the MVI tumor cells, as well as some tumor cells in other regions, were grouped into the T3 cluster. This pattern led us to identify T3 as the MVI-related tumor cell subcluster. To validate the robustness of this finding, we extended our analysis to include a combined data set of all 6 samples. Importantly, the MVI spots across the 6 samples were consistently found within the T3 subgroup, further confirming that T3 represents the MVI-related tumor cell subcluster.

Subsequently, we delved into the characteristics of these clusters within the MVI sample. To begin, Figure 3F provides a visual representation of the expression profiles of the top 5 marker genes for each cluster. Clear gene expression distinctions emerged between the tumor and normal regions. T1 and T2 clusters exhibited similar marker genes, including POSTN, COMP, and THBS2, which are markers associated with cancer-associated fibroblasts, showing heightened expression.^23^ In addtion, N1 and N2, closely related to T3, displayed several immune-related genes, such as CXCL13, CCL9, and IL7R. N3 and N4, representing normal hepatocyte areas, exhibited genes related to liver function, including ALB, FGB, FGG, and HP. N5 was enriched with B cells, featuring elevated expression of B-cell markers such as JCHAIN, IGHM, and IGHA1.^24^ T3 and T4 featured marker genes like MKI67, HIST1H1B, and AGR2, which are associated with proliferation, indicating their aggressive phenotype. Notably, SLC6A14 and CXCL5 were distinctive marker genes for T3, confirming our hypothesis that T3 acts as the progenitors of MVI.

Leveraging Slingshot, a tool designed for reconstructing putative branching transcriptional trajectories, enabled us to explore evolution among tumor clusters. Pseudotime ordering of all tumor cells organized these 4 tumor clusters into a single main branch, with T1 at the beginning and T3 and T4 at the lineage’s end (Figure 3G). Upon determining the cell cycle phase of each tumor cluster, T3 exhibited pronounced proliferation activity with a high proportion of G2M and S phase (Figure 3H). Notably, SLC6A14 and CXCL5 demonstrated transcriptional trajectories similar to that of cluster T3.

### SLC6A14 and CXCL5 were correlated with worse prognosis

**FIGURE 4:**
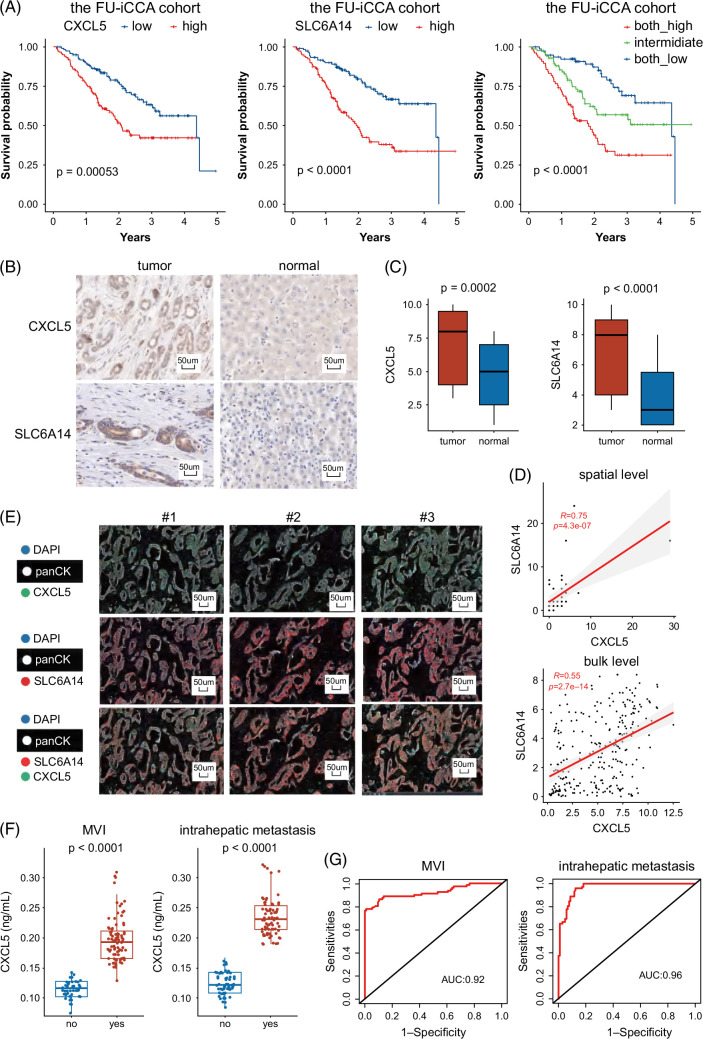
SLC6A14 and CXCL5 were correlated with worse prognosis. (A) Patients with elevated levels of SLC6A14 and CXCL5 exhibited shorter overall survival times, with those displaying high levels of both SLC6A14 and CXCL5 experiencing the poorest clinical outcomes. (B) Immunohistochemistry images of SLC6A14 and CXCL5 in our cohort consisting of 70 patients with ICC. (C) Boxplots displaying the distribution of SLC6A14 and CXCL5 in normal (n = 70) and tumor samples (n = 70). (D) SLC6A14 and CXCL5 exhibited significant correlations in spatial transcriptomics and bulk transcriptomics data. (E) The multiplex immunofluorescence performed in 20 patients with ICC demonstrated the co-location of SLC6A14 and CXCL5 in ICC tumor cells. (F) The serum level of CXCL5 was correlated with vascular invasion (n = 56) and intrahepatic metastasis (n = 74) in all samples (n = 135). (G) CXCL5 also exhibited its ability to predict vascular invasion with an AUC of 0.92 and intrahepatic metastasis with an AUC of 0.96. Abbreviation: ICC, intrahepatic cholangiocarcinoma.

Patients with elevated transcriptome expression levels of SLC6A14 and CXCL5 exhibited shorter overall survival times, with those displaying high levels of both SLC6A14 and CXCL5 experiencing the poorest clinical outcomes in the FU-iCCA cohort (Figure 4A). We also performed immunohistochemistry of SLC6A14 and CXCL5 on our in-house cohort of 70 patients with ICC. SLC6A14 and high CXCL5 demonstrated elevated expression in tumor cells compared to normal epithelial cells (Figures 4B, C). The correlation between SLC6A14 and CXCL5 reached 0.75 in the T3 cluster at the spatial transcriptomics level and 0.55 in bulk transcriptomics data of ICC, indicating a collaborative role of these 2 genes in promoting aggressiveness (Figure 4D). To validate this association at the protein level, we conducted multiplex immunofluorescence of SLC6A14 and CXCL5 on 20 samples obtained from patients with ICC. By employing panCK for annotating tumor cells, we were able to differentiate tumor cells from the adjacent microenvironment. Remarkably, we observed that SLC6A14 and CXCL5 were located in the same area within the ICC tumor cells, which aligns with the results obtained from the transcriptomics analysis (Figure 4E).

Notably, CXCL5, being a detectable chemokine in serum, raised the prospect of serving as a promising serum biomarker for MVI. Subsequently, we collected serum samples from a substantial in-house cohort comprising 135 patients with ICC, of which 56 had pathologically validated MVI and 74 had pathologically validated intrahepatic metastasis. Our results validated the heightened level of CXCL5 in the MVI group (Figure 4F). Moreover, we found CXCL5 with higher expression in the intrahepatic metastasis group. CXCL5 also exhibited its ability to predict vascular invasion with an AUC of 0.92 and intrahepatic metastasis with an AUC of 0.96 (Figure 4G).

### CXCL5 attracted immunosuppressive MRC1+ TAM in the TME

**FIGURE 5:**
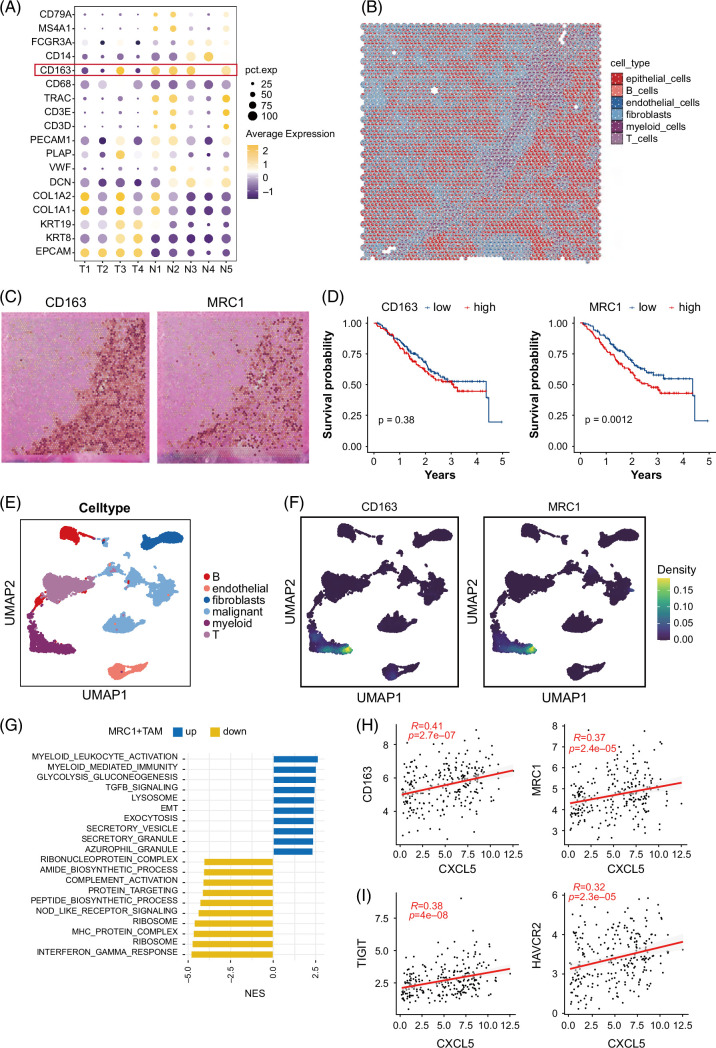
CXCL5 attracted immunosuppressive MRC1+ TAM in the TME. (A) The expression patterns of classical cell-type–specific markers among all clusters. (B) CARD algorithm was used for imputing cell-type compositions of each spot in spatial transcriptomics data. (C) The spatial pattern of CD163 and MRC1 in spatial transcriptomics data. (D) Patients with elevated levels of MRC1 exhibited shorter overall survival time, while the expression level of CD163 had no effect on clinical outcome. (E) The single-cell data set (n = 21,158) was divided into 6 main clusters: malignant cells (n = 8881), myeloid cells (n = 1555), fibroblasts (n = 2547), endothelial cells (n = 2224), T cells (n = 4302), and B cells (n = 1649). (F) Distribution of CD163 and MRC1 in single-cell data set. (G) Bar chart displaying the upregulated and downregulated pathways in MRC1+ TAM. (H) The correlation between CXCL5 and macrophage+ immune infiltration markers (CD163 and MRC1). (I) The correlation between CXCL5 and immune checkpoints (HAVCR2 and TIGHT). Abbreviations: TAM, tumor-associated macrophage; TME, tumor microenvironment.

We further proceeded to investigate the TME components around MVI and its progenitors. We first investigated the expression patterns of classical cell-type–specific markers among all clusters (Figure 5A). In total, the tumor area was accumulated with fibroblasts and endothelial cells and absent of immune cells, except T3 showing high expression of CD163, the marker of myeloid cells. The normal area displayed high immune infiltration, including B cells (CD79A and MS4A1), T cells (TRAC, CD3D, and CD3E), and myeloid cells (CD163 and CD14). We used the CARD algorithm for imputing the cell-type compositions of each spot in spatial transcriptomics data.^15^ We observed the heavy accumulation of myeloid cells in the tumor boundary and the accumulation of fibroblasts in the tumor core (Figure 5B). The spatial expression pattern of CD163 had the same spatial pattern as the composition of myeloid cells, validating the CARD deconvolution result (Figure 5C). To investigate the main subtype of myeloid cells in the tumor boundary, we compared some classical markers of myeloid subtypes and found that MRC1 had the same expression pattern as CD163 (Figure 5C). Patients with elevated levels of MRC1 exhibited shorter overall survival time, while the expression level of CD163 had no effect on clinical outcome (Figure 5D).

To accurately investigate the phenotype of MRC1, we leveraged the combination of single-cell data sets from GSE125449, GSE138709, GSE151530, and GSE181878 and identified 6 main clusters: epithelial cells, myeloid cells, fibroblasts, endothelial cells, T cells, and B cells (Figure 5E). The Violin plot exhibiting the distribution of these marker genes is shown in Supplemental Figure S2, http://links.lww.com/HC9/B158. The distribution of MRC1 only in TAM validated its role as a typical macrophage marker and was a subset of CD163+ TAM (Figure 5F). Pathway analysis unveiled increased epithelial-mesenchymal transition in MRC1+ TAM, involved in extracellular matrix remodeling and promoting tumor metastasis (Figure 5G). The immune-suppressing pathway TGFB signaling was enhanced in MRC1+ TAM. Enhanced secretory activities were observed in MRC1+ TAM, including secreting vesicles and granules. There were changes in metabolism-related pathways, including glycolysis and gluconeogenesis. Conversely, immune-related pathways (MHC protein complex, interferon-gamma response, and complement activation) were suppressed in MRC1+ TAM. We calculated the correlation of CXCL5 and macrophage+ immune infiltration markers (CD163 and MRC1) in a large cohort of patients with ICC (Figure 5H). The correlation of CXCL5 and CD163 reached 0.41, while the correlation of CXCL5 and MRC1 reached 0.37, indicating that CXCL5 might attract the MRC1+ TAM. Significantly, HAVCR2 and TIGHT were found to have a notable expression in the boundary region of MVI, indicating that they could be prospective targets for immunotherapy.^25^ The distribution of CXCL5/HAVCR2/TIGHT in single-cell data set is shown in Supplemental Figure S3, http://links.lww.com/HC9/B159. To find a more specific marker, we compared the gene expression profiles of these MRC1+ myeloid cells near MVI regions with those of MRC1+ myeloid cells located away from MVI regions. This comparison yielded a list of DEGs, with IDO1 ranking highest among them. Our findings suggest that IDO1 may serve as a more specific marker for MVI-related TAMs (Supplemental Figure S4, http://links.lww.com/HC9/B158). The elevated expression of IDO1 in MRC1+ myeloid cells near MVI regions highlights its potential role in mediating the invasive characteristics associated with these macrophages.

### CXCL5-CXCR2 and LGALS9- HAVCR2 were identified in cell-cell communication between MVI and MRC1+ TAM

**FIGURE 6:**
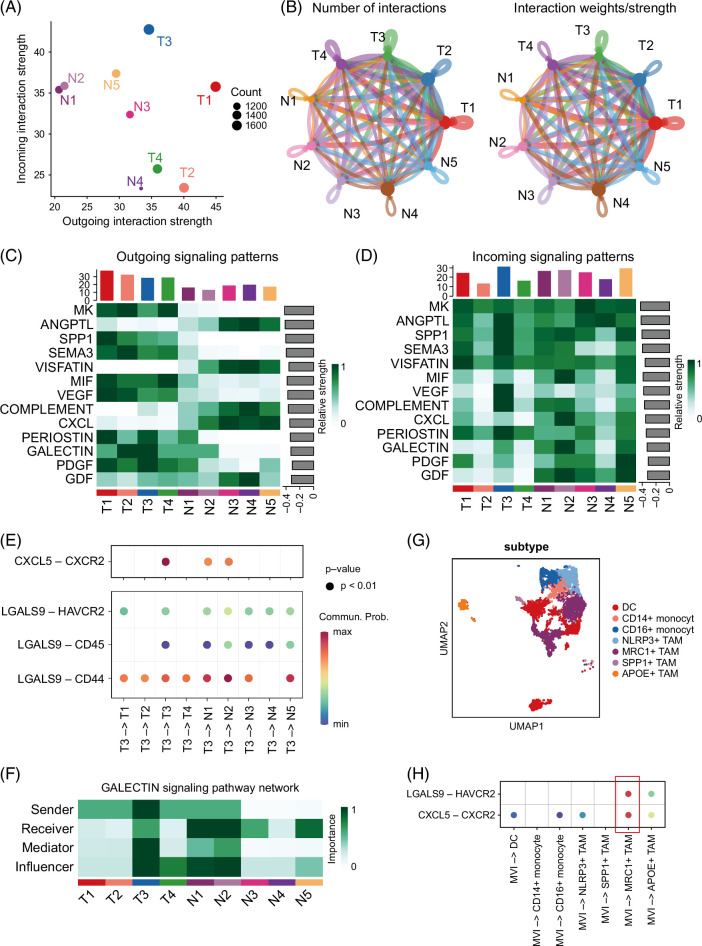
CXCL5-CXCR2 and LGALS9-HAVCR2 were identified in cell-cell communication between MVI and MRC1+macrophage. (A) The role of all clusters in cell-cell communication. (B) The intercellular communication among all clusters. (C) The outgoing signaling patterns of all clusters. (D) The incoming signaling patterns of all clusters. (E) The CXCL5-CXCR2 and ligand-receptor pairs in the GALECTIN signaling pathway signaled from T3 to other clusters. (F) The GALECTIN signaling pathway network. (G) Myeloid cells in the single-cell data set were divided into 7 subtypes: DC, CD14+ monocyte, CD16+ monocyte, NLRP3+ TAM, SPP1+ TAM, MRC1+ TAM, and APOE+ TAM. (H) The CXCL5-CXCR2 and LGALS9-HAVCR2 identified in the cell-cell communication between MVI and MRC1+macrophage. Abbreviations: MVI, microvascular invasion; TAM, tumor-associated macrophage.

We further proceeded to investigate the interactions between MVI and the local environment. T3 (the MVI and its’ progenitors) exhibited a high level of incoming and outgoing interactions (Figure 6A). The intercellular communication among all clusters was examined (Figure 6B). We then overview the outgoing and incoming signaling pathways, where the tumor area and normal area displayed distinct outgoing signaling patterns (Figures 6C, D). Tumor area activated various proliferation and metastasis-related signaling pathways (MK, SPP1, SEMA3, MIF, VEGF, PERIOSTIN, and PDGF) and immunosuppression-related signaling pathway (GALECTIN). In contrast, normal areas activated various immune-related signaling pathways (COMPLEMENT and CXCL) and lipid-related signaling pathways (ANGPTL).

The release of CXCL5 from T3 cells targets the expression of CXCR2 in N1 and N2 cells (Figure 6E). The CXCL5/CXCR2 axis has been implicated in promoting tumor development, angiogenesis, and the activation of host cells.^26^ Furthermore, the GALECTIN signaling pathway was detected in the interaction between T3 and adjacent N1 and N2 cells, where LGALS9 interacted with CD44, CD45, and HAVCR2 (Figure 6E). LGALS9, a constituent of the galectin family, primarily functions as an inhibitor of the immune system.^27^ Elevated levels of HAVCR2 expression facilitate the development of tumors, the rapid growth of cancer cells, and their ability to invade surrounding tissues by inhibiting the actions of immune cells.^28^ HAVCR2 binds to LGALS9, which triggers inhibitory pathways that reduce T-cell activities responsible for inducing immunological tolerance.^29^ T3 was identified as the main source of the GALECTIN signaling pathway, whereas N1 and N2 located in the surrounding region acted as recipients and agents that influenced the GALECTIN signaling (Figure 6D).

To instigate the role of CXCL5-CXCR2 and LGALS9-HAVCR2 in cell-cell communication between MVI and MRC1+ TAM, we evaluated their crosstalk at the single-cell level. First, we divided the myeloid in the single-cell data set into 7 subtypes: DC, CD14+ monocyte, CD16+ monocyte, NLRP3+ TAM, SPP1+ TAM, MRC1+ TAM, and APOE+ TAM (Figure 6G). Then the CXCL5-CXCR2 and LGALS9-HAVCR2 were identified in the cell-cell communication between CXCL5+ SLC6A14+ tumor cells and MRC1+ macrophages (Figure 6H).

## DISCUSSION

The surgical management of ICC faces significant challenges due to high rates of disease recurrence. One contributing factor is MVI, a well-known predictor of disease recurrence and poor clinical outcomes after surgery. In this study, we conducted a comprehensive investigation into the phenotype of MVI in ICC. Our findings identified CXCL5 and SLC6A14 as robust predictors of MVI. Notably, serum CXCL5 emerged as a promising noninvasive diagnostic tool. Moreover, our research revealed that MVI and its progenitor cells possess the ability to recruit MRC1+ TAM while concurrently excluding CD8 T cells, establishing an immune-resistant microenvironment. These insights provide valuable information for refining the understanding of MVI pathology and may pave the way for improved strategies in the surgical management of ICC by considering the role of MVI and its associated immune microenvironment.

Our primary objective was to characterize marker genes associated with MVI and elucidate their phenotype. Notably, CXCL5 and SLC6A14 emerged as robust predictors of MVI. SLC6A14, functioning as a Na+/Cl−-coupled amino acid transporter, is recognized for transporting 18 of the 20 essential amino acids.^30^,^31^ Depletion of SLC6A14 has been linked to amino acid starvation, suppressing epithelial-mesenchymal transition–induced metastasis in cancer. Previous research has underscored the potential of SLC6A14-targeted nanoparticles in resetting amino acid metabolism in cancer cells for enhanced anticancer therapy.^32^,^33^ CXCL5 within the TME is primarily produced by tumor cells in specific cancers like HCC, breast cancer, and ICC.^34^,^35^,^36^,^37^ Studies indicate that CXCL5 induction is driven by IL-17A or IL1 in the TME, endowing tumor cells with an invasive phenotype.^34^,^38^ In addition, CXCL5 plays a vital role in the creation of pre-metastatic environments and is increased in metastatic lung tissue, directing the movement of cancer cells through the CXCL5/CXCR2 pathway.^39^ In a mouse xenograft model, Zhou et al^40^ demonstrated that CXCL5 increased the proliferation and metastasis of ICC.

In addition, our work examined how MVI and their progenitors affect the regulation of the TME, revealing their influence on tumor immunity. The close proximity of MVI to cancer cells indicated the possibility of juxtacrine interactions. MRC1+ TAMs, which is a subset of TAM, have the ability to control the number of defective T cells by using cytokine/chemokine signaling. The presence of MRC1+ macrophages is directly associated with the proportion of CD8-PDCD1 cells. The MRC1+ macrophages exhibited an abundance of functions associated with chemokine-mediated signaling, as well as the regulation of complement, exocytosis, extracellular matrix, and cell adhesion. Patients who have a large amount of TAM in their tumor tissues are much more likely to develop advanced clinical-stage disease, experience metastasis, develop medication resistance, and have a low chance of survival.

In addition, our study unveiled the interaction between MVI and the MRC1+ macrophage. Prior research has indicated that the CXCL5/CXCR2 axis has the ability to enhance tumor development and angiogenesis, hence facilitating the infiltration and activation of host cells.^41^ The CXCR2+ cell populations that are recruited play a crucial role in the malignant development of malignancies and can be targeted for cancer treatment.^26^ Due to the importance of the CXCL5/CXCR2 axis in different types of malignancies, multiple small molecule inhibitors have been created to target this axis.^42^ Adding a CXCR2 inhibitor has been proven to improve the effectiveness of treatment when used together with AB680 and a PD-1 inhibitor.^31^ Moreover, the levels of other checkpoint molecules, such as TIM3, TIGIT, and CTLA4, were significantly associated with CXCL5 in multiple forms of cancer. TIM3 engagement, when it binds to LGALS9 or CEACAM1, leads to the death of CD8+ T cells or their functional exhaustion, respectively.^43^ Preclinical studies have provided insights into the therapeutic possibilities of inhibiting TIM3 in conjunction with anti–PD-1 antibodies. These findings highlight the possible ways in which MVI hinders the immune system’s ability to remove cancer cells and speeds up the growth of tumors.

Our study has numerous significant strengths when compared to earlier research. This study provides the initial thorough analysis of MVI in ICC, exploring its spatial arrangement and considerably enhancing our understanding. The successful identification and validation of MVI indicators establish a basis for their dependable detection and targeted therapies. However, it is essential to recognize specific constraints in our study. The lack of patients who received immunotherapy in our group emphasizes the necessity for additional research on the involvement of MVI in immunotherapeutic settings. In addition, direct validation through in vitro or in vivo experiments is essential to establish the interaction between MRC1+ TAMs and CXCL5+SLC6A14+ tumor cells. Once the animal model is established, we will construct ICC cell lines with either knockout or overexpression of CXCL5 and SLC6A14. These cells will then be implanted subcutaneously to allow tumor growth. Upon harvesting the tumors, multiplex immunofluorescence will be performed to examine changes in the TME, with a particular focus on MRC1+ TAMs. We believe this comprehensive approach will provide the necessary validation and insight into the interaction between MRC1+ TAMs and CXCL5+SLC6A14+ tumor cells.

## CONCLUSIONS

This study demonstrated the phenotype and related spatial patterns of MVI in ICC, identifying CXCL5 and SLC6A14 as potential markers and therapeutic targets.

## Supplementary Material

SUPPLEMENTARY MATERIAL
