# Supplementary material for: Spatial analyses revealed CXCL5 and SLC6A14 as the markers of microvascular invasion in intrahepatic cholangiocarcinoma
Source: Hepatol Commun. 2024 Dec 11;9(1):e0597. doi: 10.1097/HC9.0000000000000597 (PMC11637745; doi:10.1097/HC9.0000000000000597)
Supplement: SUPPLEMENTARY MATERIAL [file hc9-9-e0597-s001.pdf]

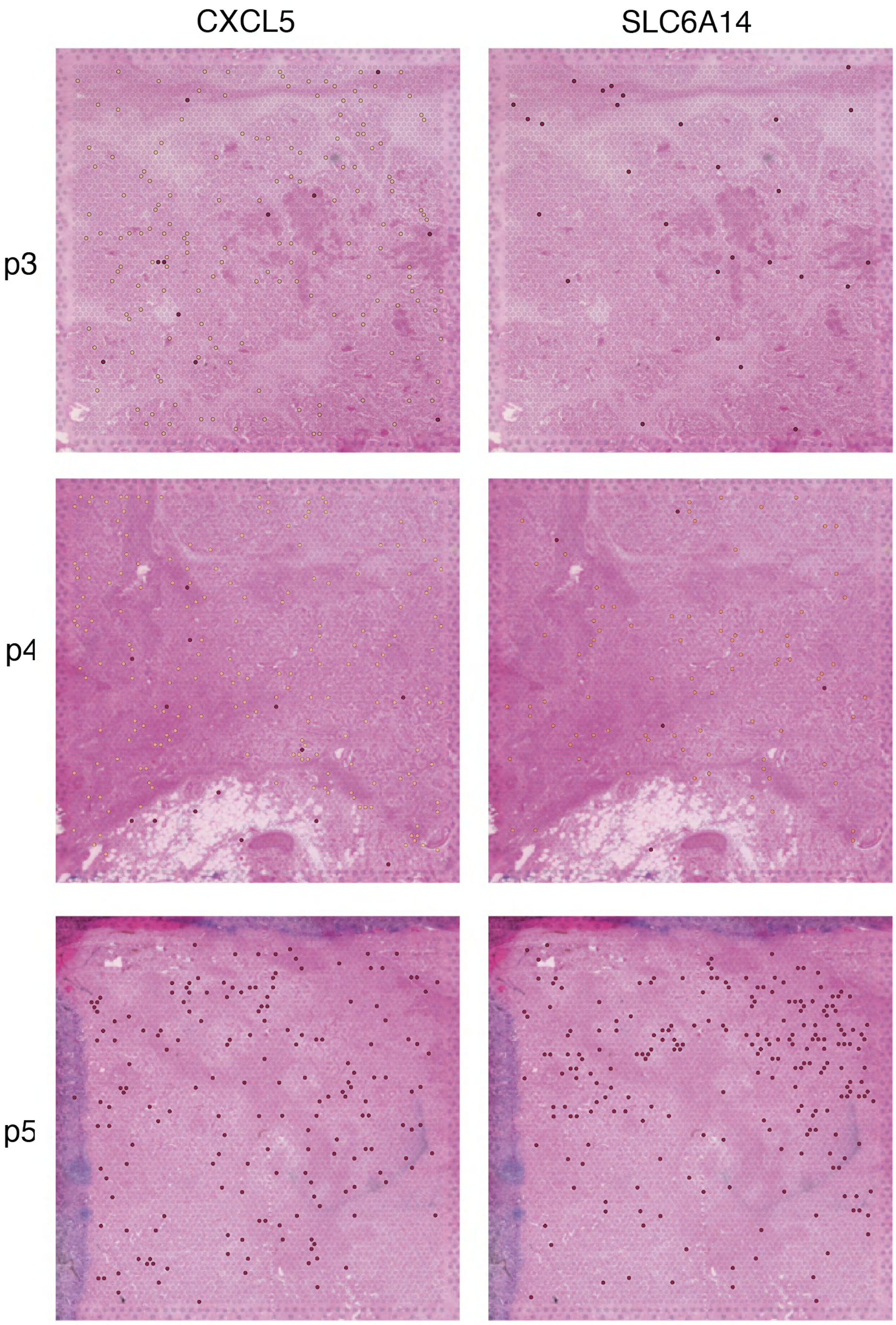


Figure S1. in the remaining three samples without metastasis tendencies, SLC6A14 and CXCL5 displayed low expression levels

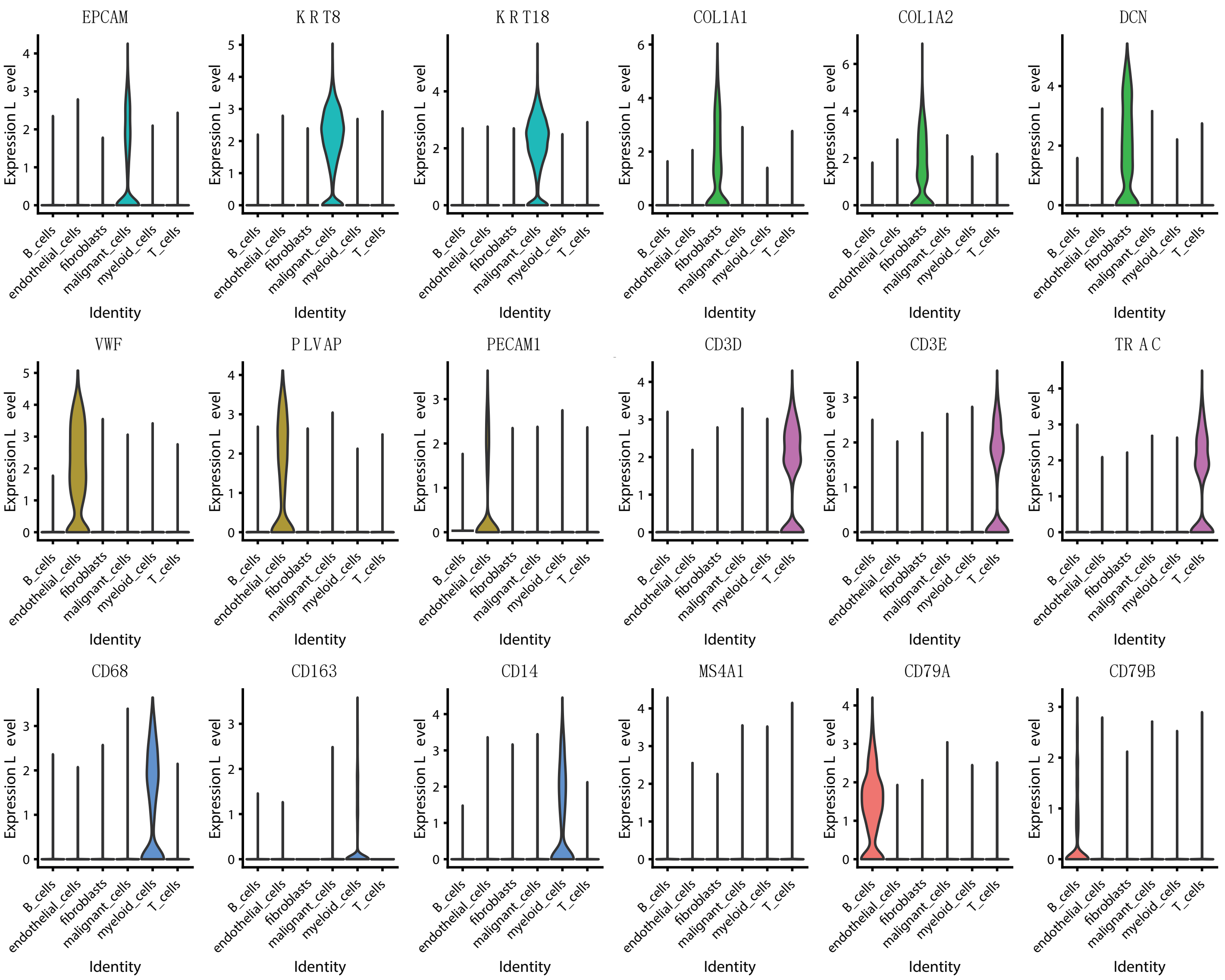


Figure S2. The distribution of the marker genes: epithelial cells (EPCAM, KRT8, KRT19), fibroblasts (COL1A1, COL1A2, DCN), endothelial cells (PLVAP, VWF, PECAM1), T cells (CD3D, CD3E, TRAC), B cells (MS4A1, CD79A, CD79B), and myeloid cells (CD14, CD163, CD68).

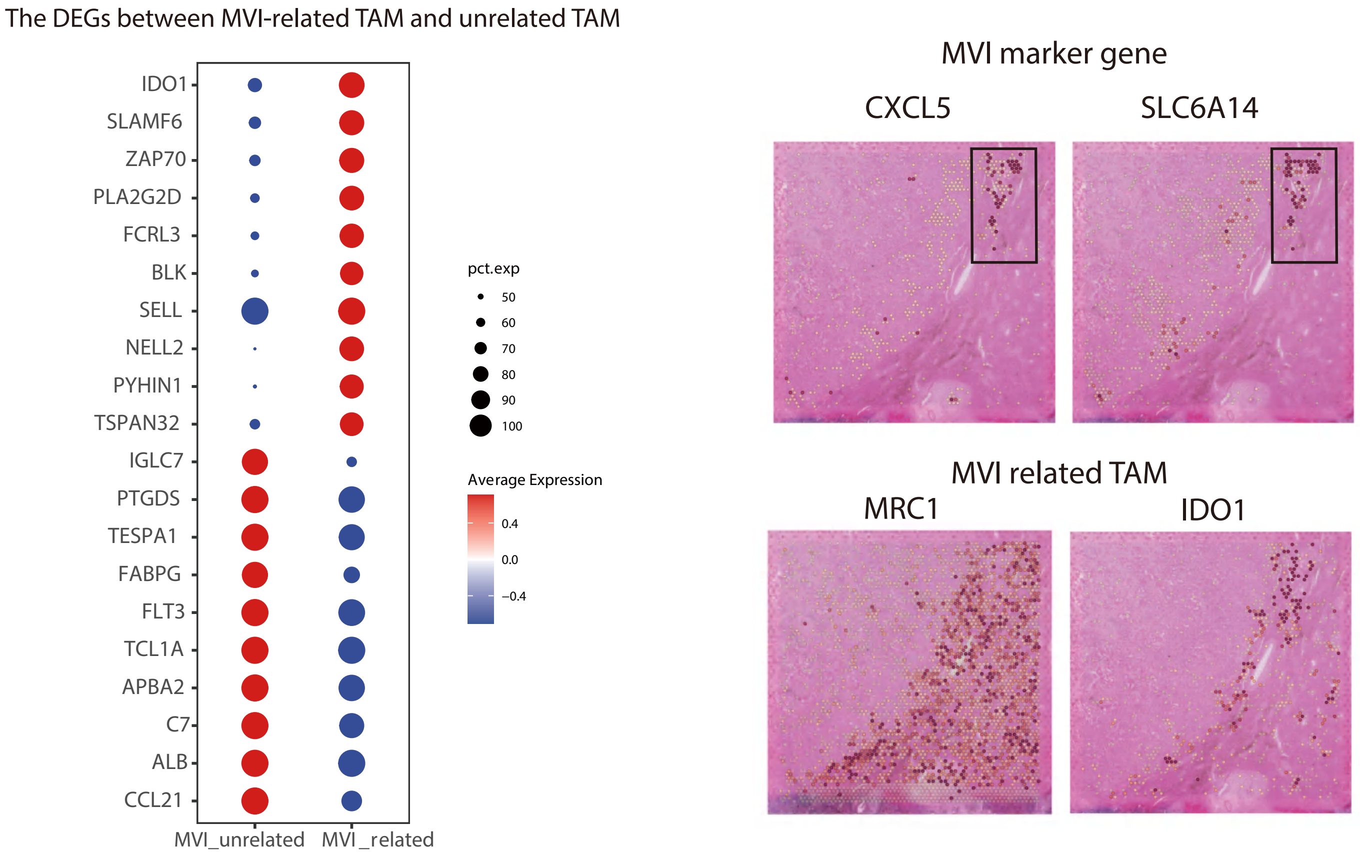


Figure S4. IDO1 may serve as a more specific marker for MVI-related TAMs.

Table S1. The marker gene of STAS tumor cells.

| | | | | |
|---|---|---|---|---|
| MUC5AC | VILL | FAM3D | NQO1 | CDC42EP5 |
| S100P | MT-ND3 | CYP3A5 | GATA6 | STARD10 |
| TFF2 | LYZ | TINAGL1 | PTGDS | HIST2H2BE |
| TSPAN8 | SULT1E1 | SOX9 | MUC13 | ABHD2 |
| LEFTY1 | TSPAN1 | B3GNT3 | GATM | PRSS8 |
| MUC6 | TRNP1 | FER1L6 | JUNB | NDNF |
| PSCA | SOD3 | CST3 | ALDH1A1 | POF1B |
| LGALS4 | B3GALT5 | CLU | A2M | CLDN23 |
| CA2 | SMIM24 | ADIRF | TMBIM1 | KLF5 |
| GABRP | ADH1C | ARL14 | KCNE3 | CREB3L1 |
| REG4 | EPS8L3 | PLA2G10 | SFTPC | ASAP2 |
| SPINK1 | CAPN9 | USH1C | EYA2 | HNF4A |
| MUCL3 | CAMK2N1 | GALNT5 | COL27A1 | SLC4A4 |
| ANXA10 | KRT17 | GOLM1 | GLUL | ANXA13 |
| MSLN | PKDCC | TRAK1 | TRIM7 | MS4A8 |
| TFF1 | VSIG2 | BAIAP2L2 | CTSE | KIF12 |
| AGR2 | ATP2A3 | GPR35 | SPINK13 | IGFBP7 |
| TFF3 | GMDS | HPGD | LMO4 | TMC5 |
| MUC5B | SDCBP2 | SHROOM3 | SLC12A2 | GDF15 |
| HMGCS2 | SLC44A4 | FMOD | MMP1 | |
| MUC3A | PROM2 | DUOX2 | GPT | |
| MIA | CYSTM1 | VWF | METTL7A | |
| CLDN18 | IGHG1 | IGHG3 | MYOM1 | |
| PGC | FXYD3 | GALNT12 | METRN | |
| PHGR1 | TOB1 | LTBP2 | TRIM29 | |
| ERN2 | ZFP36L2 | ITM2C | LBH | |
| PLAC8 | LCN2 | HIST1H2AC | FOXQ1 | |
| CHIT1 | CXCL5 | RETREG1 | PIK3C2G | |
| GPX2 | NPDC1 | SH3BGRL2 | ZG16B | |
| GCNT3 | ALDOB | IGHM | TSPAN3 | |
