# Supplementary figures and images for: Spatial analyses revealed CXCL5 and SLC6A14 as the markers of microvascular invasion in intrahepatic cholangiocarcinoma
Source: Hepatol Commun. 2024 Dec 11;9(1):e0597. doi: 10.1097/HC9.0000000000000597 (PMC11637745; doi:10.1097/HC9.0000000000000597)

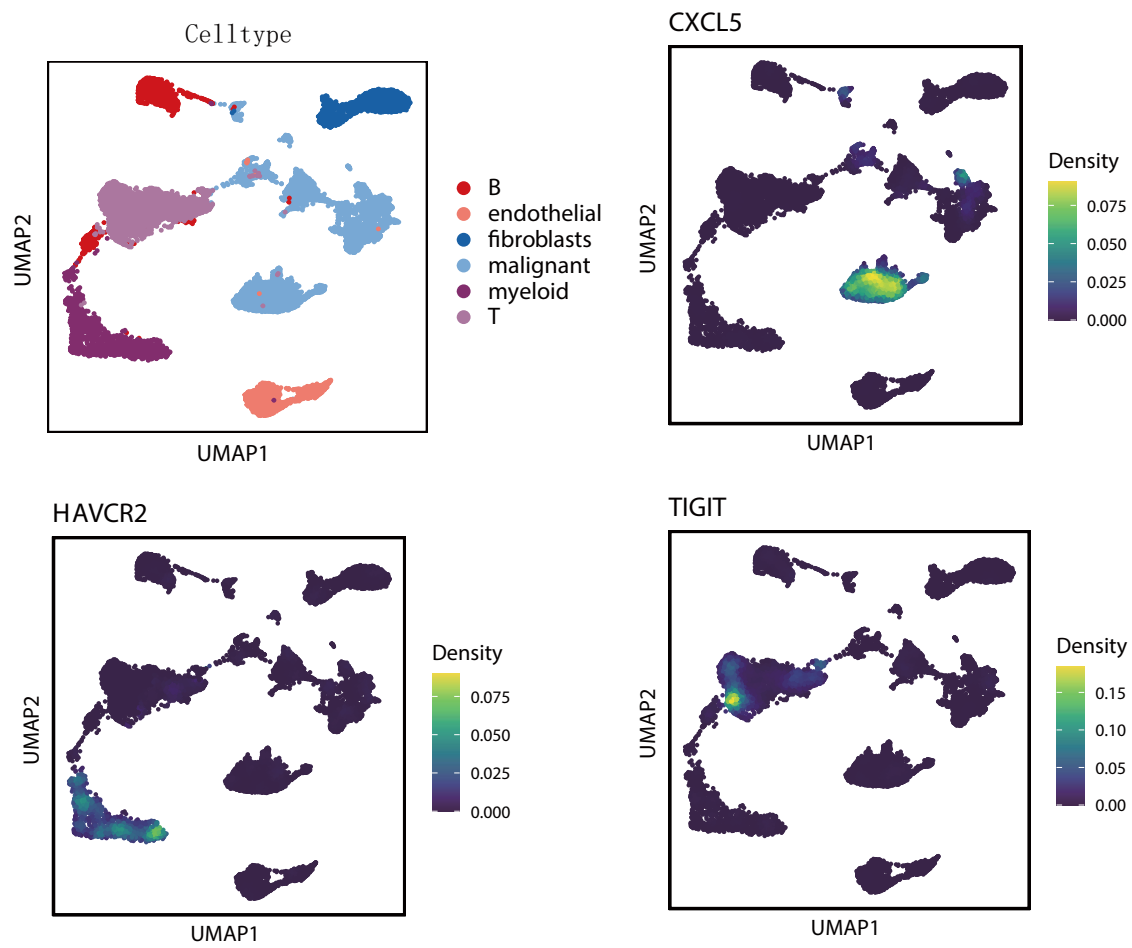

Figure S3. The distribution of CXCL5/HAVCR2/TIGIT in single-cell dataset

Supplement: SUPPLEMENTARY MATERIAL [file hc9-9-e0597-s002.pdf]
